# Evaluation the reactivity of a peptide-based monoclonal antibody derived from OmpA with drug resistant pulsotypes of *Acinetobacter baumannii* as a potential therapeutic approach

**DOI:** 10.1186/s12941-022-00523-5

**Published:** 2022-06-30

**Authors:** Omid Yeganeh, Mahdi Shabani, Parviz Pakzad, Nariman Mosaffa, Ali Hashemi

**Affiliations:** 1 Department of Microbiology, North Tehran Branch, Islamic Azad University, Tehran, Iran; 2 Department of Immunology, School of Medicine, Shahid Beheshti University of Medical Sciences, Tehran, Iran; 3 Department of Microbiology, School of Medicine, Shahid Beheshti University of Medical Sciences, Tehran, Iran

**Keywords:** *Acinetobacter baumannii*, Antibiotic resistance, Monoclonal antibody (mAb), Outer membrane protein A (OmpA), Passive immunization, Antimicrobials

## Abstract

**Background:**

*Acinetobacter baumannii* is an opportunistic and antibiotic-resistant pathogen that predominantly causes nosocomial infections. There is urgent need for development nonantibiotic-based treatment strategies. We developed a novel monoclonal antibody (mAb) against a peptide of conserved outer membrane protein A (OmpA) and evaluated its reactivity with different pulsotypes of *A. baumannii*.

**Methods:**

Peptide derived from *A. baumannii* OmpA was conjugated to keyhole limpet hemocyanin and injected into BALB/c mice. Splenocytes of immunized mice were fused with SP2/0 myeloma cells followed by selection of antibody-producing hybridoma cells. After screening of different hybridoma colonies by ELISA, one monoclone was selected as 3F10-C9 and the antibody was tested for reaction with five different *Acinetobacter* pulsotypes that were resistant to carbapenem antibiotics. The affinity constant was measured by ELISA. The ELISA, western blotting, indirect immunofluorescence (IFA), and in vitro opsonophagocytosis assays were used to evaluate the reactivity of generated mAb.

**Results:**

The anti-OmpA antibody reacted with the immunizing peptide and had a high affinity (1.94 × 10^−9^ M) for its antigen in the ELISA. Specific binding of mAb to OmpA was confirmed in Western blot. IFA assays revealed that mAb recognized specific OmpA on the pulsotypes. Opsonophagocytosis assays showed that the mAb increased the bactericidal activity of macrophage cells. The antibody function was higher in the presence of serum complement.

**Conclusions:**

The peptide-based mAb demonstrated optimal performance in laboratory experiments which may be appropriate in investigation on OmpA in *Acinetobacter* pathogenesis and development of passive immunization as a novel therapeutic approach.

## Background

*Acinetobacter baumannii* has become a life-threatening pathogen associated with community-acquired and nosocomial infections, particularly among immunocompromised patients who have a weakened immune system. This opportunistic bacterium has the ability to accumulate drug resistance mechanisms, and also an augmentation in the number of antibiotic-resistant strains reduces effective treatment and increases mortality [1]. The growing resistance to beta-lactam drugs, carbapenems, and even colistin antibiotics complicates an effective antibiotic therapy and raises the need for new strategies to prevent and treat infections caused by *A. baumannii* [2, 3]. The acquired resistance profiles including multidrug-resistant (MDR), extensively drug-resistant (XDR) and pandrug-resistant (PDR) bacteria are often responsible for healthcare-associated infections which usually lead to higher medical costs, prolonged hospital stays, and increased mortality throughout the world [4]. Hereupon, the healthcare institutions must be aware of infections caused by members of the genus *Acinetobacter*. It has been authenticated that neutrophils, macrophages, complement system, and specific antibodies are necessary to effective control and elimination of these bacterial pathogens [5, 6]. Data concerning the impact of MDR *A. baumannii* are insufficient and controversial. There are currently no approved vaccine offering significant protective efficacy against acute *A. baumannii* infection [7, 8]. Beyond that, compared to other bacteria, a limited number of antibiotics are able to be effective against *Acinetobacter* while showing low toxicity to human cells [9]. There seems to be an urgent need to implement infection control measures and antimicrobial stewardship programs to prevent the further spread of drug resistant *Acinetobacter* species and even postpone the increasing resistance in other bacteria.

Despite an antibiotic or a small peptide, whose function is simply to bind and modulate a target, the antibodies possess the other capabilities due to their Fc region including opsonophagocytic activity, agglutination process, and activating the complement system. In this regard, the antibodies are essential in cases such as, triggering immunity against *A. baumannii*, induction of protective mechanisms, blocking of bacterial attachment to the epithelial cells, the opsonization process, and the complement-dependent degradation of the bacteria [6, 10]. Considering the important role of antibodies in humoral immunity, monoclonal antibody (mAb) could be designed to interact with specific targets and provide complementary protection as an immunotherapy or passive immunization [11, 12]. Outer membrane protein A (OmpA), one of the major outer membrane proteins in gram-negative bacteria, is an essential virulence factor that mediates bacterial biofilm formation, eukaryotic cell infection, antibiotic resistance, virulence, and immunomodulatory mechanisms [13]. OmpA is a class of β-barrel integral membrane proteins settled in bacterial outer membrane, whose molecular mass ranges from 28 to 36 kDa [14]. In the past few years, studies have shown that the amino acids of this protein from a variety of clinical isolates are highly conserved in evolution (> 89%) sharing minimal homology with the human proteome [15, 16]. Therefore, OmpA has been considered as an antigenic candidate in development of mAbs against *A. baumannii* [17, 18].

Considering the tertiary structure of proteins, anti-peptide antibodies are not expected to react with all their respective proteins. However, scientific evidence exists that shows antibodies against synthetic peptides could interact with their corresponding proteins [19]. The mAbs that target OmpA may open new possibilities for immunotherapy by providing an excellent cellular targeting and could be useful for studying the physiological functions of this evolutionarily conserved protein. More accurate techniques will be used in the future clinical trials to identification and even biotherapy of this opportunistic nosocomial pathogen.

This study aimed to evaluate the reactivity a peptide-based mAb with OmpA protein in antibiotic resistant pulsotypes of *A. baumannii* and survey whether the conserved surface-exposed OmpA in these different pulsotypes of *A. baumannii* holds the potentials to be an antigen candidate for passive immunotherapy in the future.

## Materials and methods

### Ethics statement

This work was carried out under the supervision of the institutional research ethics committee of Islamic Azad University, Science and Research branch, Tehran, Iran (Approval ID: IR.IAU.SRB. REC. 1398. 064).

### Preparation of OmpA-derived peptide as an immunogen

Based on previous in-silico design and bioinformatics analysis, a 27 amino acid peptide (VTVTPLLLGYTFQDSQHNNGGKDGNLT) at N-terminal region located at 24–50 position derived from OmpA of *A. baumannii* was designed and used as a safe and suitable immunogen for mice immunization [20]. As previously described, the OmpA antigenic epitopes were predicted using different tools with the highest score and based on hydrophobicity, antigenicity, flexibility, mobility, accessibility, polarity, exposed surface, and coils. Then, among five OmpA consensus epitopes, one of the novel synthetic peptides was selected [peptide 1 (amino acids located in the 24–50 position of the OmpA protein)] that had been elicited higher immune responses [20].

### Peptide conjugation to carrier protein

The synthetic peptide was conjugated to bovine serum albumin (BSA) and keyhole limpet hemocyanin (KLH) (Sigma, St. Louis MO, USA) using glutaraldehyde 1% as linker separately and simultaneously utilizing the same buffer systems and methods, as described [6]. As, the KLH is a large aggregating protein and has a high molecular weight (4.5 × 10^5^ to 1.3 × 10^7^ Da) it does not penetrate well into very fine particles of polyacrylamide gel in the SDS PAGE [21]. Indeed, high molecular mass complexes remain at the top of the electrophoresis gel. For this purpose, a carrier protein such as BSA with lower molecular weight (about 67 KDa) was prepared simultaneously using the same method and used to evaluate the accuracy of conjugation. In this way, 1 mg (400 µl) of immunogen peptide (First BASE laboratories, 604944-X, Malaysia) and 1 mg of dissolved KLH (500 µl) were mixed together. Then 70 μl of distilled water and 30 μl of glutaraldehyde 1% were added to the mixture to obtain a final concentration of 1 mg/ml for the peptide-KLH. Similarly, to prepare the peptide-BSA, 0.5 mg of dissolved BSA (250 µl) was mixed with 0.5 mg of the peptide (200 µl) along with 50 μl of distilled water and 15 μl of glutaraldehyde. The peptide–KLH conjugate was used to mice immunization and the peptide-BSA was used for conjugation assessment.

### Mice immunization procedure

In this study, which is a new experience in the production of monoclonal antibody (mAb) against drug-resistant *Acinetobacter baumannii*, three female BALB/c mice were used for an immunization model. The mice were healthy, young and free of infection and were purchased from the Royan Institute, Iranian academic center for education, culture and research (ACECR). Mice were about 6 weeks old and were injected 5 times intraperitoneally with 25 μg of the OmpA-peptide on days 0, 21, 35, 49, and 63 [22]. Each mouse was injected with a total of 125 μg of peptide antigen. In addition, to induce an acceptable immune response, the first injection was carried out using complete Freund’s adjuvant (Sigma-Aldrich, Saint Louis, USA), while the other five injections were performed using incomplete Freund’s adjuvant (Sigma-Aldrich, Saint Louis, USA). Ten days after the last injection, mice were tail-bled and the sera were assayed for antibody molecules against antigenic peptide in an indirect ELISA test. One mouse with a highest titer of anti OmpA-peptide was selected for further steps. Finally, three days before the cell fusion, 20 μg of peptide-KLH in PBS were injected intravenously to the well immunized mouse [6].

### Evaluation of mice immune response by enzyme-linked immunosorbent assay (ELISA)

Wells of the ELISA plate (Greiner-Bio-One, Italy) were coated with 50 µl OmpA-derived peptide (10 µg/ml) overnight at 4 °C. After washing three times with PBS-Tween 20 (0.05%) (Sigma-Aldrich, St Louis, Mo, USA), the plate was blocked with 2.5% BSA at 37 °C for one hour and also at 4 °C for three hours. Wells were then washed three times. To evaluate the immune response of mice, different dilutions of mice serum taken from the tail vein were added to the coated wells (50 µl/well), starting from titer of 1:500 and at 37 °C for one hour [23]. After washing, 50 µl/well of HRP-conjugated rabbit anti-mouse Ig (1:1000; Avicenna Research Institute, Tehran, Iran) was added and incubated for one hour at 37 °C. After washing, 50 µl of tetramethylbenzidine (TMB) (DNAbiotech Co, Iran, Cat No: DB9510) was added to each well and the plates were incubated at room temperature (RT) in the dark. After about 12 min, 15 µl of stopping solution (20% H_2_SO_4_) was added to each well. The optical densities (OD) of the reactions were measured at 450 nm by an ELISA reader system (Anthos 2020, Salzburg, Austria).

In order to evaluate the amount of produced antibodies, ELISA tests were performed in duplicate at each stage of blood sampling. The mouse with the highest titer of specific antibody was considered for cell fusion and hybridoma generation. To screen for the antibody production by the hybridoma cells, the same procedure was performed on the culture supernatants.

### Cell fusion process

One of the mice with the highest titer of specific antibody was chosen and used for fusion process. The mouse was euthanized by exposure to CO_2_ and placed on a clean portable plate. Using sterile instruments such as scissors and forceps, the spleen was removed and the spleen cells were isolated. In order to develop suitable hybridoma cells, mouse myeloma SP_2_/0 cells and splenocytes of the immunized mouse were washed with pre-warmed RPMI medium (pH: 7.2) and then mixed in a ratio of 1:5 respectively. After rinsing the cell mixture, pre-warmed (37 °C) 50% polyethylene glycol (PEG) 1500 (HybriMax, Sigma-Aldrich, USA), as a non-ionic hydrophilic polymer, was added to the cell pellet slowly with continuous mixing [24]. After pouring 20 ml of the wash medium, cells were centrifuged at 20 °C for 5 min at 1000 rpm (or 500 G). Cell pellets containing fused cells were then cultured in the RPMI-1640 medium (Gibco by life technologies, USA). Also, some factors for cell culture such as sterility, pH, nutrients, and proper temperature were considered. As a selective medium, hypoxanthine-aminopterin-thymidine (HAT) supplemented medium (Sigma, USA) was added to the wells and only hybridoma cells were allowed to grow [24]. For better growth of hybridomas, fetal bovine serum (FBS) (Gibco, USA) was used at concentrations of 10% or 20% (v/v) as an enrichment in the culture medium [25]. The cell culture supernatants were then tested for the mAb evaluation by ELISA as a mentioned above. Hence, among the grown hybrid cells, the mono clones capable of producing mAbs were screened. Then, a mono clone with freshly condition and without possible contamination was selected as 3F10-C9 clone.

### Isotype determination of mAb by capture ELISA

Goat anti-mouse IgG1, IgG2a, IgG2b, IgG3, IgA, and IgM subclass specific antibodies including Lambda (λ) and Kappa (κ) at 1/1000 dilution (Sigma-Aldrich-ISO2 MSDS, USA), with 50 µl/well, were adsorbed on to the wells of a microtitre ELISA plate (SPL, Korea). After blocking and washing similar to the above explanation, the supernatant of the 3F10-C9 clone was added in the amount of 100 µl to each well. Also, PBS 1× was added instead of 3F10-C9 mAb to each well as a negative control. After performing the ELISA steps similar to the previous description, the isotype of 3F10-C9 mAb was determined.

### Determination of affinity constant

The affinity constant (K_aff_) of mAb from the clone 3F10-C9 was determined by non-competitive enzyme immunoassay method as previously described [26]. All reactions were done in a sealed (to prevent evaporation) microtitre polystyrene plate (Maxisorp, Nunc, Denmark) with all reaction volumes of 50 µl. Briefly, an ELISA plate, pre-coated with five different concentrations of peptide (5000, 2500, 1250, 625, 312.5 ng/ml), were then separately incubated with serial dilutions of the 3F10-C9 mAb. After performing the ELISA test steps as described above, the amount of mAb adherent to the peptide antigen in each well was reflected by the measured OD_450_. The use of serial dilutions of mAb resulted in sigmoid curves of OD versus antibody concentrations. Sigmoid curves were plotted using the OD_450_ values against the logarithmic antibody concentrations. The half of maximum OD (OD-50) on each curve was determined at 450 nm. Then, antibody concentration was assigned on the X-axis corresponding to OD-50 of each antigen curve. [Ab]_t_ and [Ab′]_t_ were the measurable total antibody concentrations in the wells at OD-50 and OD-50' for plates coated with [Ag] and [Ag′], respectively. K is the antigen–antibody affinity constant in l/mol (M^−1^). The K_aff_ was then calculated using the following equation [26]:

$$Kaff=\left(n-1\right)/2 \left(n \left[Ab^{\prime}\right]t-\left[Ab\right]t\right)$$

where,

$$n=\left[\mathrm{Ag}\right]t/\left[\mathrm{Ag}^{\prime}\right]t$$

### Affinity purification of generated mAb

3F10-C9 mAb was affinity purified using a column of pre-activated resin, CNBr-activated sepharose 4B (GE Healthcare, Uppsala, Sweden), conjugated to the OmpA-peptide. The elution was performed using 0.1 M glycine–HCL (pH: 2.7). The eluted antibody was dialyzed overnight against PBS 1× (pH: 7.2) at 4 °C, and the antibody reactivity was measured by ELISA as described above.

### Preparation of bacterial samples

Bacterial samples were collected from carbapenem-resistant *A. baumannii* isolates in hospitals that have been previously studied [27]. In the previous published work, these isolates harbored resistance genes such as OXA-23, OXA-24 and OXA-58 and subsequently were analyzed for possible presence of resistance indices including Ambler class A, metallo-β-lactamases (MBLs), carbapenem-hydrolysing class D β-lactamases, extended spectrum β-lactamases like (bla_TEM_, bla_PER_, bla_GES_) and insertion sequence of ISAba1. The genetic relatedness between the isolates was previously analyzed using the pulsed-field gel electrophoresis (PFGE) method [27]. Among different clonal pulsotypes of *Acinetobacter baumannii*, we randomly received five different isolates including pulsotypes A, B, C, D and E (kind gifts from Dr. Zohreh Ghalavand) and subjected them to various experiments.

### Western blot analysis

A single colony of each bacterial isolates was harvested from the bacterial culture and then centrifuged [5000 rpm for 5 min]. Bacterial lysates were prepared as previously described with modifications [28, 29]. Briefly, bacterial pellets were suspended in 1 ml lysis buffer (lysis buffer: 150 mM Sodium chloride, 2 mM EDTA, 1 mM NaF, 40 mM Na_4_P_2_O_4_, 0.1% SDS, 50 mM Tris pH 7.4, 1% glycerol, and 1% Triton X-100). The suspension was boiled for 5 min and sonicated three times for 20 s (Ultrasonic Apparatus XO-650, Xianou, Nanjing, China). The supernatant of sonicated proteins from bacteria were considered as the native OmpA lysates, which were prepared by breaking *Acinetobacter* pulsotypes.

As stated, OmpA of *A. baumannii* is closely similar to *Escherichia coli* OmpA and OprF of *Pseudomonas aeruginosa* [30]. The supernatants from sonicated *E. coli* (ATCC 25922) and *P. aeruginosa* (ATCC 27853) lysates were used as quality control to evaluate antibody reactivity with the samples loaded in the gel. Accordingly, bacterial lysates were loaded at 15 μg on each well of 10% bis–tris gel with SDS running buffer. After electrophoresis, resolved proteins were transferred from the gel onto Immobilon-PVDF blotting membrane (Millipore, Billerica, Massachusetts, USA). The membrane was blocked overnight at 4 °C with 5% non-fat dry milk (Sigma, Millipore, USA) in PBS-Tween 20 (0.05%). After gentle washing with PBS-Tween, 3F10-C9 mAb (20 μg/ml) was added to the membrane and incubated for 90 min at RT. The membrane was washed extensively with PBS-Tween and incubated with HRP-conjugated rabbit anti-mouse IgG (Avicenna research institute, Tehran, Iran) (1:2000) for 1 h at RT, followed by washing and developing with DAB (3,3′-Diaminobenzidine) detection system (Thermo Scientific, PI34002, USA).

### Indirect immunofluorescence assay (IFA)

To detect surface binding of *A. baumannii* by 3F10-C9 mAb, an indirect immunofluorescence assay (IFA) was developed as previously described with modifications [11]. About 1.5 × 10^6^ of each *Acinetobacter* pulsotype (isolates A to E) and also *E. coli* were prepared and washed twice with PBS pH 7.2, and then centrifuged at 5000 rpm for 5 min. After washing, bacterial cells were incubated with 100 µl of purified 3F10-C9 mAb (20 µg/ml) as the primary antibody for one hour at 37 °C. After washing twice with PBS-Tween, the subject microtubes were incubated with 100 µl of fluorescein isothiocyanate (FITC) conjugated sheep anti-mouse immunoglobulin as secondary antibody (Dilution 1:500; Avicenna research institute, Tehran, Iran) for 45 min. After washing with PBS-Tween, 50 µl of each sample was placed on the slide and covered with coverslip to observe microscopically under ultraviolet light.

### Opsonophagocytic killing assay

The opsonization assay was performed to examine the antibody which act to coat the bacterial cell wall and prepare it for ingestion [11, 16]. Five pulsotypes of *A*. *baumannii* were cultured overnight in Luria–Bertani broth medium at 37 °C, then passaged to mid-log growth, rinsed, and suspended in a sterile PBS 1×. Murine macrophage RAW 264.7 cells were cultured at 37 °C in 5% CO_2_ in DMEM/F-12 growth medium (Gibco, USA) with 10% FBS. The RAW 264.7 cells were activated by four days of exposure to 1 µg/ml lipopolysaccharide (LPS) (Sigma-Aldrich, L2880, USA). LPS-differentiated RAW cells were transferred from the culture flask to a microtiter plate (~ 2 × 10^6^ cells/ml, 80 µl/well). Then, five isolates of *A. baumannii* were added distinctly to each well of the plate (~ 1 × 10^6^ CFU/ml, 10 µl/well). The 3F10-C9 mAb at a concentration of 50 µg/ml were then added to each well (10 µl/well) for 6 h at 37 °C. For complement studies, non-immune mouse serum was added to the wells (10 µl/well) in both of active and heat-inactivated forms in order to create conditions with complement and without complement, respectively. For quality control, *Escherichia coli* ATCC 25922 strain was used as a gram-negative bacterium that has a relative structural similarity to *Acinetobacter* [31]. (Notably, if available, the *A. baumannii* knock-out of the OmpA gene can be used for the quality control). In addition, as a negative control, a non-specific monoclonal antibody with the IgG isotype was used instead of the 3F10-C9 mAb in the presence and absence of the serum complement. After 6 h incubation of mixtures with gentle shaking, the supernatant of each well was removed and quantitatively plated in Mueller–Hinton agar. Then, the number of bacteria colony forming units (CFU) of each well were counted after an overnight culture and followed by calculation the approximate rate of bacterial mortality [32].

### Statistical analysis

All statistics analyses were run using GraphPad Prism 8 software. Evaluation of the relationship between bacterial killing and type of opsonin substances (mAb or Isotype control antibody), in two groups with complement and without complement, was studied using the chi-square test separately for different pulsotypes of bacteria. Differences were considered significant if the p-value was < 0.05.

## Results

**Fig. 1:**
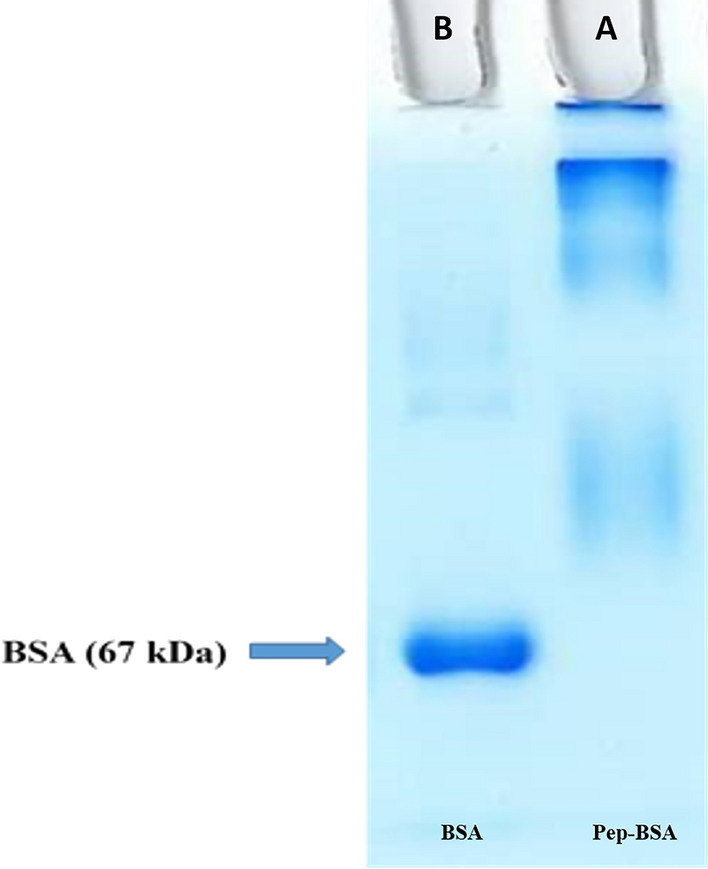
Conjugation of OmpA-peptide to BSA carrier protein. The peptide-BSA conjugate shows a wide smear on the gel that is caused by conjugation of peptide molecules to BSA as compared to the sharp band of unconjugated BSA. Lane **A** Uprising mobility of peptide-BSA reveals that total immunizing peptides were conjugated to BSA molecules. Lane **B** Pure BSA, which appears as a single band in the range of 67 KDa of SDS-PAGE gel. Due to multimeric and high molecular mass of KLH, it is not feasible to run the KLH conjugate on SDS-PAGE. In this regard, BSA conjugate was used for efficacy of conjugation

**Fig. 2:**
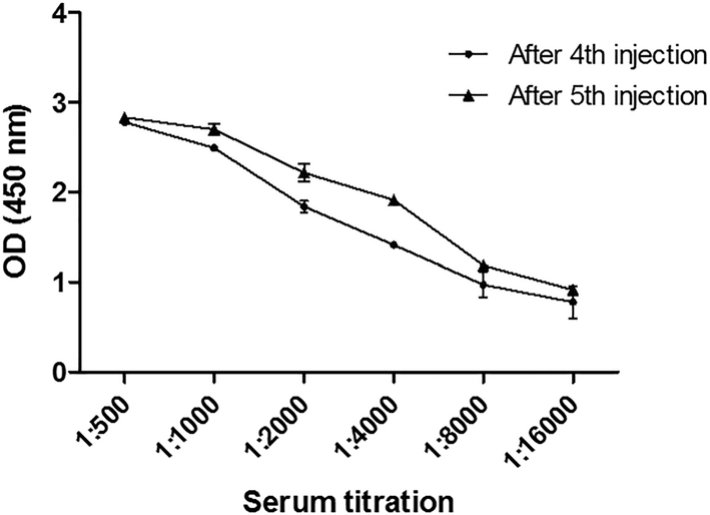
Serum titration diagram of an immunized mouse at separate intervals. Wells were coated with OmpA-peptide and the serum titration was performed from dilution of 1:500 to 1:16,000. As a negative control, phosphate-buffered saline was added to the last well of each ELISA strip column. Obtained data demonstrated that the mouse was suitable for cell fusion. The mean and standard deviation were determined for each point. Tests were performed in duplicate

**Fig. 3:**
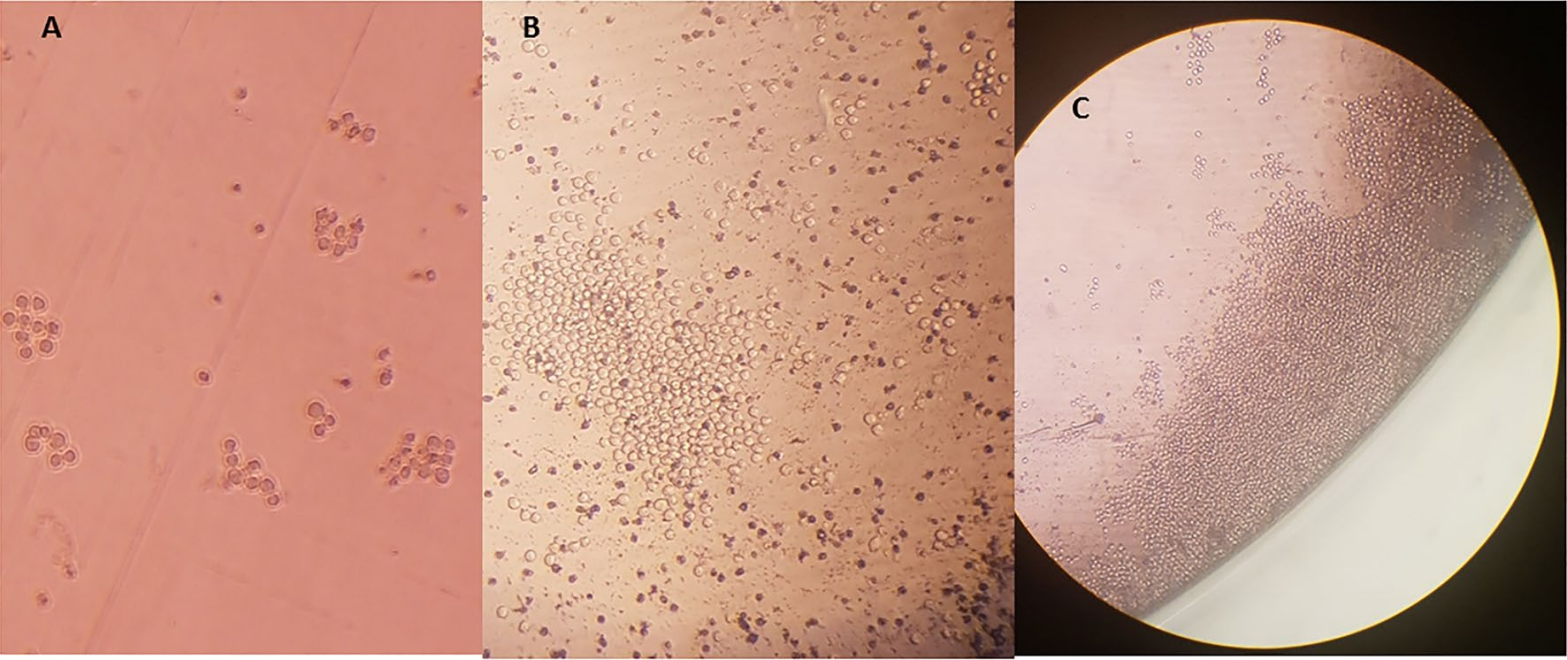
Stages of hybridoma cell growth. MAbs can be renewably generated, once a single clone of hybridoma is developed. The produced mAbs are homogenous and consistent. **A** Initial growth of hybridoma cells after survival in the HAT selected medium. **B **One monoclone in a growing form. **C** The monoclone in the highly proliferated form

The use of antibodies against leading antigens which aids to diagnosis and protective immunity is thought to be effective. We employed a peptide-based antibody generation for producing mAb against the OmpA antigen which had high expression levels in *Acinetobacter*. In this respect, BSA and KLH were selected as the carrier proteins to be conjugated with OmpA-specific peptide. Correct conjugations of the peptide to carrier proteins were assessed by the SDS-PAGE electrophoresis. Indeed, change in mobility shift of the peptide-BSA conjugate on SDS-PAGE gel demonstrated the efficiency of conjugation (Fig. 1). Accordingly, peptide-KLH was used to immunize BALB/c mice and peptide-BSA was applied to confirm our conjugation. Following mice immunization, the titers of anti-OmpA antibodies were measured in mice sera by ELISA test. The results of the serum titration by ELISA showed that one of the treated mice was well immunized after the fifth injection (Fig. 2). After fusion between splenocytes of immunized mouse and myeloma SP2/0 cells, supernatants of growing hybridoma cells were screened based on reactivity with OmpA-peptide by ELISA. Among several positive clones, one monoclone, designated as 3F10-C9, had a strong reactivity with OmpA. After the growth and proliferation of the cells of 3F10-C9 clone, subsequent tests were carried out with the mAb from this clone (Fig. 3).

### Isotype determination and affinity measurement

**Fig. 4:**
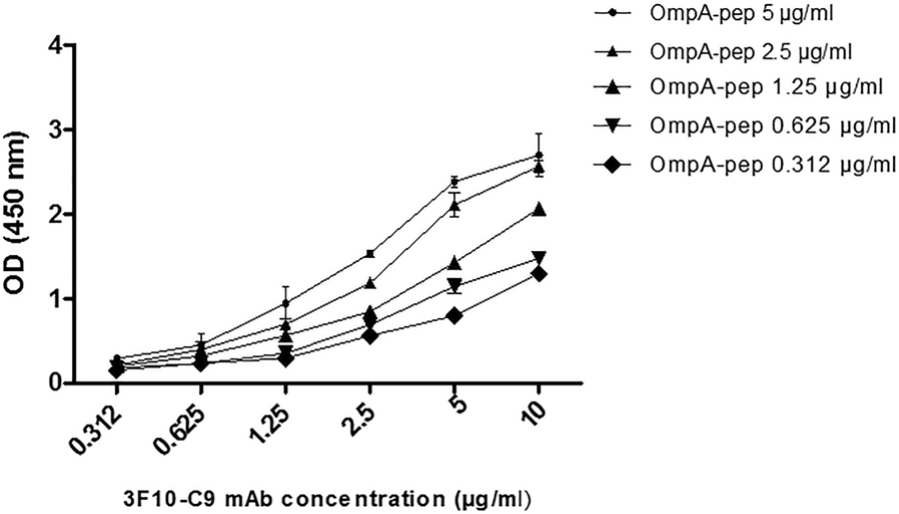
Affinity constant (Kaff) of mAb 3F10-C9 for peptide-OmpA by ELISA. To determine the exact affinity constant, the affinity constant test was performed twice. Representative curves employed for extrapolation of K_aff_ of the 3F10-C9 mAb

The ELISA method was applied for isotype determination of produced anti-OmpA mAb. In this regard, isotype of mAb 3F10-C9 was determined to be IgG1 with kappa (κ) light chain. The affinity constant (K_aff_) of 3F10-C9 mAb was also calculated by home-made ELISA as described in the methods section. In this regard, different dilutions of the mAb were separately applied to five different peptide concentrations. Sigmoid curves were plotted to represent the relationship of OD_450_ value versus logarithmic mAb concentration in five different antigen concentrations (Fig. 4). The affinity constant was then calculated 1.94 × 10^−9^ Mol.

### Western blot analysis

**Fig. 5:**
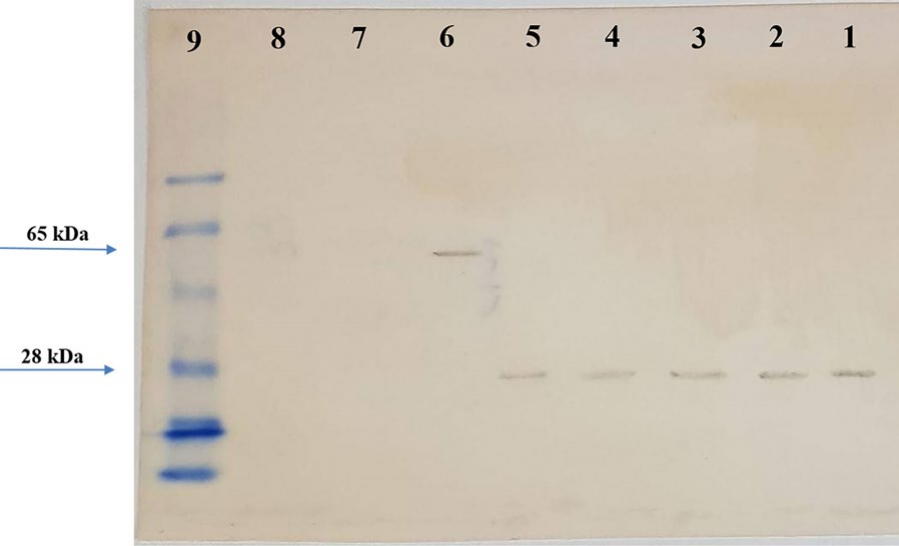
Western blot analysis of the mAb reactivity. OmpA protein is considered as the main nonspecific slow porin of *A. baumannii*. Since OmpA appears to be homologous with the *E. coli* OmpA and *P. aeruginosa* OprF, the bacterial lysates containing OmpA or similar protein were resolved in the 10% acrylamide gel and then exposed to the 3F10-C9 mAb to evaluate the antibody reactivity. Lanes **1**–**5** Lysates from pulsotypes A to E of *A. baumannii*, Lane **6** BSA-conjugated peptide, Lane **7** *E. coli* lysate, Lane **8** *P. aeruginosa* lysate, Lane **9** SeeBlue pre-stained protein standard Marker

In Western blotting, DAB substrate enabled chromogenic detection of HRP-activity at the site of OmpA protein. Accordingly, Western blot analysis indicated that 3F10-C9 mAb appropriately reacted with the OmpA present on isolates of A to E of *A. baumannii* and with OmpA-peptide in the BSA-peptide column. As a result, Western blot experiment showed that 3F10-C9 mAb recognized OmpA molecules around 28 kDa molecular weight. Further, the mAb detected the peptide molecules around 65 KDa which were conjugated to the BSA carrier protein (Fig. 5). There was no reaction between the 3F10-C9 mAb and lysates of *E. coli* and *P. aeruginosa*.

### IFA assay and mAb recognition for OmpA

**Fig. 6:**
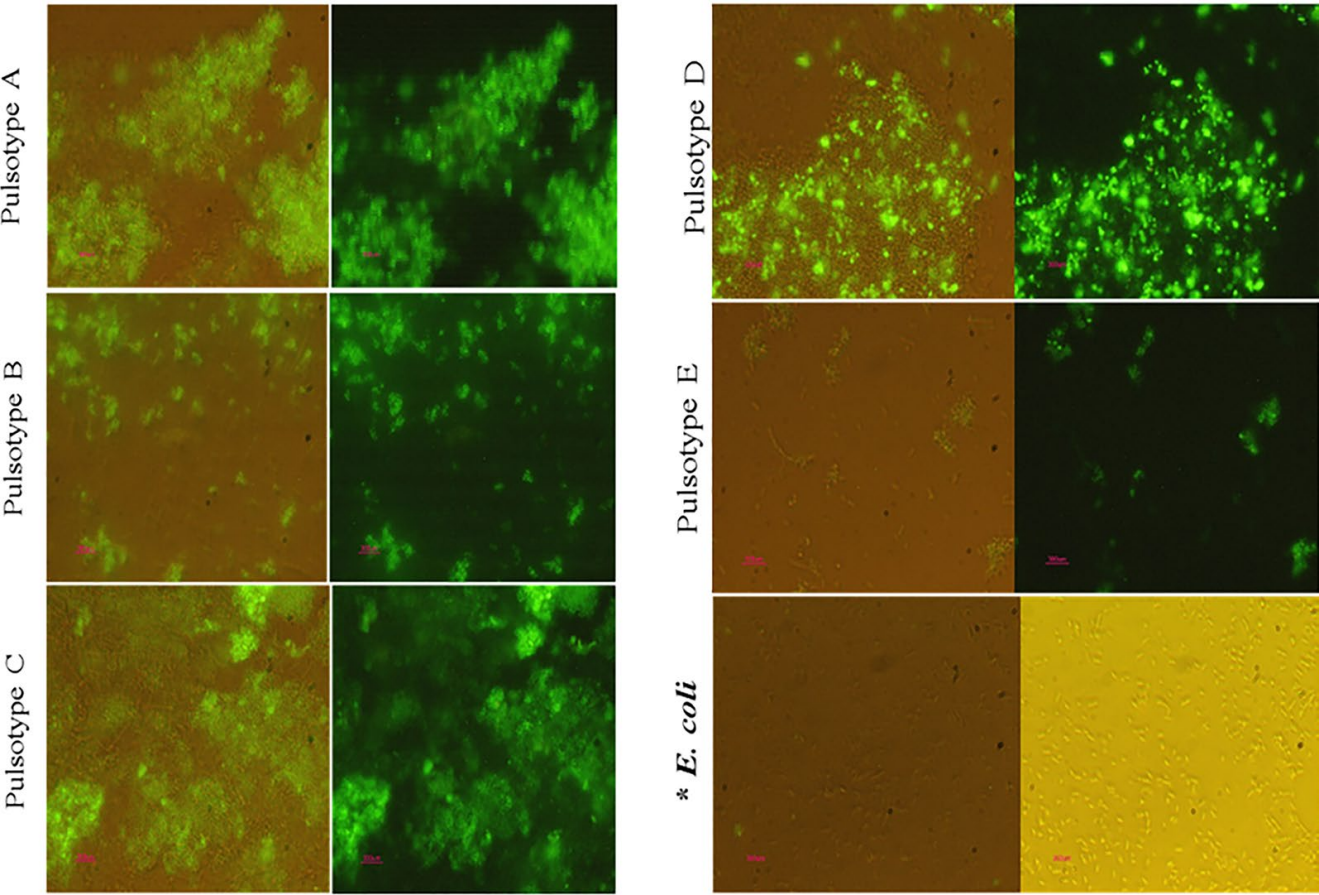
Detection of *Acinetobacter* with peptide-based mAb by IFA immunostaining. In each row, the bacteria are exposed in both normal and UV light modes in one shot of a microscopic image. (Magnification: 40×). The point areas with fluorescent light in each part of the figure represent that OmpA proteins are expressed superficially on the *Acinetobacter* pulsotypes and the peptide-based mAb by binding to exterior OmpA was able to identify the pulsotypes. *Escherichia coli as a gram-negative bacterium with similar OmpA proteins that have not been detected by the mAb in IFA immunostaining. It did indicate that the produced peptide-based mAb was specific to OmpA of *Acinetobacter*

The reactivity of 3F10-C9 mAb with native OmpA on bacterial surface was investigated by IFA assay. Acquired images revealed that the antibody recognized extracellular OmpA molecules on five various pulsotypes (A-E) of *A. baumannii*. No reaction was observed with the mAb and *E. coli* cells (Fig. 6), which was probably due to the specificity of the antibody.

### The 3F10-C9 mAb increases macrophage-mediated bactericidal activity in vitro

**Fig. 7:**
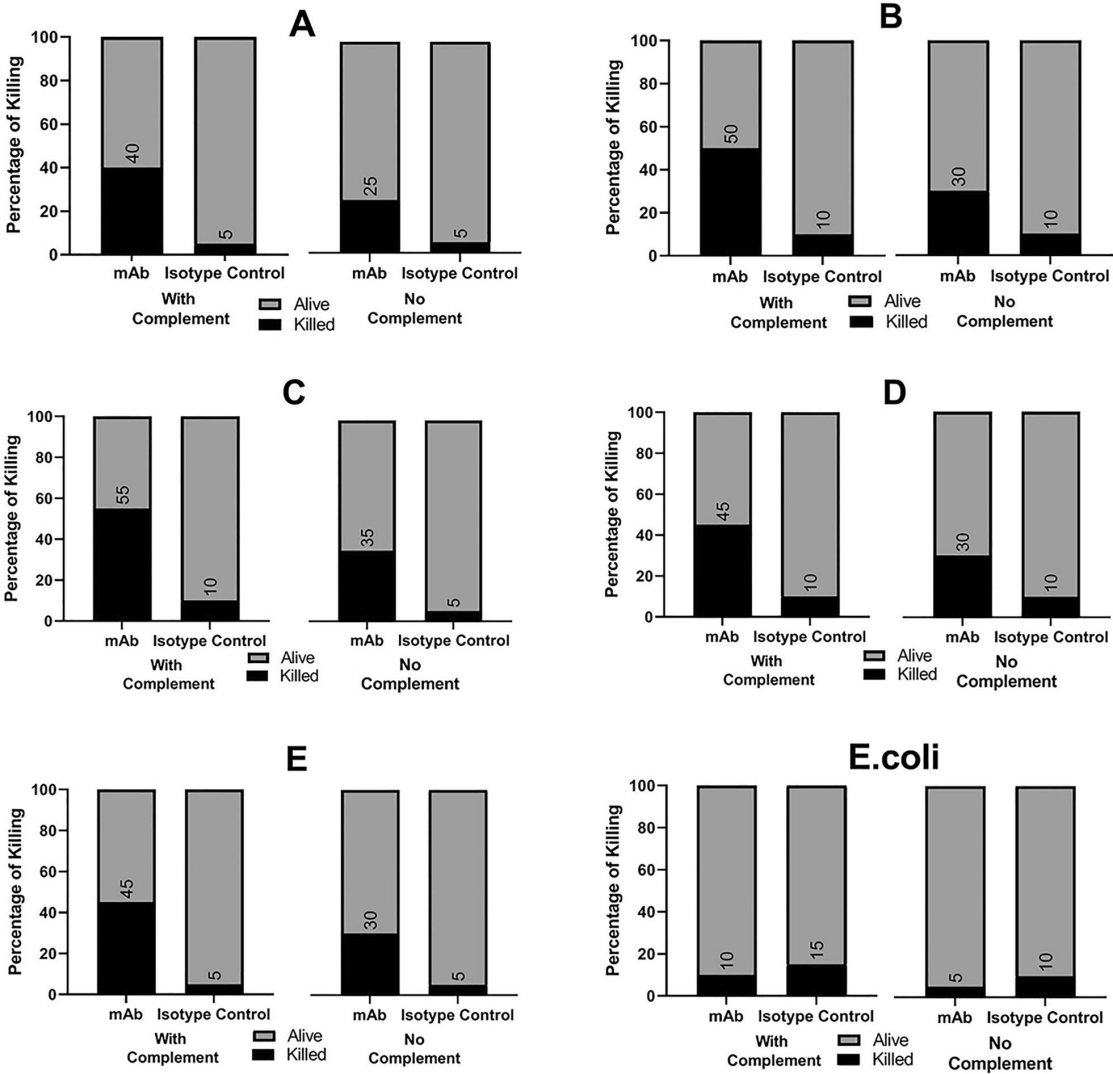
Evaluation of opsonophagocytic effects of the 3F10-C9 mAb. *A–**E** represent the opsonization results for pulsotypes **A**–**E** of *A. baumannii*

Compared to IgG1 isotype control, the opsonization with 3F10-C9 mAb significantly increased bactericidal activity of macrophage cells against the various *Acinetobacter* pulsotypes (*p* < 0.001), and also the approximate rate of bacterial death increased significantly in the presence of serum complement factor. However, the isotype control in the presence or absence of complement did not have a significant effect on bacterial mortality. In addition, the evidence demonstrated that the pulsotypes were better opsonized and consequently more harvested than *E. coli* bacteria in the presence of 3F10-C9 mAb and macrophage cells. Indeed, the opsonization of *E. coli* with the 3F10-C9 mAb and isotype control under different conditions (with and without complement) was not significant. Therefore, a specific chemical interaction between antibodies and candidate targets could be effective in the opsonization process (Fig. 7). These results showed that the increased macrophage-mediated bactericidal activity in the presence of 3F10-C9 mAb could support the hypothesis of the therapeutic potential of the specific antibodies against bacteria. Antibodies that specifically target a biomarker such as OmpA or others, without bactericidal activity, may desist from triggering bacterial evolution pressure and could be used alone or combined with other antibacterial compounds to apply synergistic effects.

## Discussion

In recent years, *A. baumannii* is believed to have accounted for a considerable proportion of conditioned nosocomial infections, such as ventilator-associated pneumonia (VAP), catheter associated urinary tract infections, and infections of bloodstream, wound, or surgical wards in the hospitals [33]. These hospital-acquired infections are common across all parts of the world and are often resistant to most antibiotics which usually lead to increased treatment costs, overuse of antibiotics, and antibiotic resistance [34]. Lack of antibiotics that remain active against refractory isolates of *A. baumannii* suggests possible roles of vaccination and passive immunotherapy as alternative strategies to fight these nosocomial infections. In vaccine development programs, some antigenic cell components such as, inactivated whole cells, outer membrane vesicles (OMVs), outer membrane complexes (OMCs), outer membrane proteins (OMPs), and even capsular polysaccharides have been shown to be effective in protection against *A. baumannii* challenge in animal models [35, 36]. However, the solubility, variability and immunogenicity of these antigens indicate some limitations to delivery of protective vaccines [7]. On the other hand, an active vaccine may cause undesirable or deleterious immune responses that can affect the safety and efficacy of the vaccine in clinical trials [37]. In this case, the use of passive immunotherapy including injections of approved monoclonal antibodies is recommended, especially when there is no opportune time for active immunotherapy [11]. Bacterial surface sight is widely used to identify and screen of antigenic determinants, following the production of high-affinity antibodies that were able to detect surface antigens [38, 39]. It has been shown that immunization with the outer membrane proteins of microorganisms stimulates and induces protective immune responses, and antibodies could passively protect laboratory animal models [16, 40, 41]. OmpA has been reported to be involved in cases such as facilitating the bacterial acclimatization to environmental stresses, reaction with the epithelial cells, and induction apoptosis in host cells [42, 43]. Despite potential levels of OmpA as a strong immunogen, other studies have shown that OmpA is soluble and biologically active when recovered from the supernatant of *Acinetobacter* culture, but insoluble when expressed as a recombinant protein [29]. On the other hand, molecular interactions indicated that OmpA was naturally toxic to host cells and the clinical isolates of *A. baumannii* overexpressing OmpA ascertained higher morbidity and even mortality in patients [44, 45]. Therefore, we decided to identify the surface peptides related to the N-terminal part of the OmpA protein and then used it as a safer antigen to produce peptide-based mAbs.

Since, most of the proteins within the *Acinetobacter* proteome are uncharacterized, the study of OmpA protein and the identification of extra-loop immunogenic peptides is a rewarding endeavor. In view of this, it has been stated that OmpA-designed peptides could serve as an appropriate candidate in diagnostic kits and predicted to be more immunogenic which may provide more permanently protection against *A.baumannii* in immunotherapy models [6, 20, 46]. A peptide fragment is thought to be a soluble antigen which could be used as a valuable biomaterial in research fields such as biological experiments, antibody engineering, medical diagnostics, drug targeting, and biotherapy. Likewise, an epitope or antigenic determinant is defined as the portion of a larger antigen that generally binds to an antibody secreted by B-lymphocytes [19].

With the bioinformatics technique and peptide design principles, an epitope with desirable properties could be prepared in quantity in order to raise specifically its protective immune responses. This suggests that a properly-selected peptide from OmpA could serve as a feasible tool with low toxicity for antibody production which is able to protect the host against *A. baumannii*. It has been shown that immunization with a conserved region of *Acinetobacter* virulence factor can increase biofilm degradation and counteract *Acinetobacter* virulence [47].

For this purpose, we picked over the 27-amino acid peptide “VTVTPLLLGYTFQDSQHNNGGKDGNLT” located at 24–50 position of OmpA at N-terminus region for immunization of mice models. Because there may be limitations to the use of a simple peptide in the immunogenicity that may result in low immune stimulation, the selected peptide was attached to the high molecular weight carrier protein-KLH and admixed with the Freund’s adjuvant. In line with the present study, Wang-Lin et al. produced anti-OmpA mAbs by immunizing mice with recombinant OmpA and Freund’s adjuvant to enhance the immune response and stated that; due to the diversity of clinical strains of *A. baumannii* and their resistance to antibiotics, discovering the therapeutic potentials of passive immunization is an essential matter [48].

Due to the small and weak body of the treating mice, it has been suggested that the number of immunogenic injections be reduced. The Fig. 2 shows that titers of the serum antibody after the fifth injection are not much different from the previous injection. In the present study, we stopped mice immunogenicity after five injections. In immunological experiments, a high affinity reaction with an affinity constant between 10^–9^ and 10^–12^ is required because the antigen and antibody binding should not be easily separated during the washing and/or testing process. In the present study, we produced a monoclonal antibody that had a high affinity constant about 1.94 × 10^9^ M^−1^ (Fig. 4). It seems that the 3F10-C9 could distinguish the antigenic peptide as a non-conformational epitope on the OmpA molecules of *A. baumannii* (Fig. 5).

In the present study, the 3F10-C9 mAb showed positive reactions with OmpA of several antibiotic-resistant *A. baumannii* pulsotypes in the Western blot and IFA tests. These experiments may be useful in evaluating antibodies for their diagnostic potentials. It is worth to mention that for clarifying the generated mAb’s reactivity with different pulsotypes of *A. baumannii*, we selected 5 distinct pulsotypes (A, B, C, D and E). These samples were characterized in the previous published work [27]. In IFA assay, the green fluorescent light reflected from the surface of pulsotype E was weaker than other strains of *A. baumannii* (Fig. 6). It may be related to the position of protein molecules on surface of the pulsotype E. Furthermore, it may even be possible that the washing, preparation or sampling steps of the desired pulsotype were different.

Opsonophagocytosis assays showed in-vitro that mAb had highly efficient bactericidal activities on clinical *A. baumannii* pulsotypes which was associated with a complement-dependent effect (Fig. 7). The effect of 3F10-C9 mAb on pulsotypes B and C was slightly higher than other pulsotypes, although this difference was not significant. It may be depended on the number and distribution of OmpA proteins on surface of the pulsotypes.

Consistent with the results of this study, T. W. Loehfelm et al. showed that 6E3 mAb against biofilm-associated protein (Bap) detected its epitope in whole-cell lysate of *A. baumannii* 307-0294 in Western blot assay and noted that the Bap antigen is an accessible surface antigen and contains surface-exposed epitopes [49]. Weiwei Huang et al. developed passive immunizations against an outer membrane protein with molecular weight of about 22 kDa (Omp22) which had the potentials to be a candidate target for opsonophagocytic killing assay and said that the effects of in-vitro opsonophagocytosis by their antiserum were partly complement-dependent [32]. Baig et al. in their study in Canada, produced two mouse mAbs, F241G3sc2 and F241G6sc2, against *A. baumannii* ATCC 19606, but the specific target of the antibodies or the exact epitope of developed mAbs on tested bacterium was almost unknown [18]. Another study in Tokyo, Japan, reported the generation of mAb against OmpA of *E. coli*. However, it was not a peptide-based antibody and was likely to interact with other Enterobacteriaceae [40]. In the study by Luo et al. anti-OmpA antibodies enhanced opsonophagocytic killing of the *A. baumannii* but did not enhance complement-mediated killing [16]. It may be due to the serum resistance of some *Acinetobacter* isolates by binding of H-factor in serum to outer membrane proteins. Russo et al. stated that the K1 capsular polysaccharide from *A. baumannii* could be as a passive immunization target and mAb 13D6 could enhance the in vitro neutrophil-mediated bactericidal activity; however, only 13% of the *A. baumannii* strains had the K1 capsular polysaccharide in that study [50]. Given that, it appears that the polysaccharide capsules are not present in all strains of the *A. baumannii.*

Despite many advances in the production of new antibiotics, we are still witnessing mutations and the resistance of microorganisms that threaten human health. Therefore, efficient design and production of mAbs can be used as an alternative method or in combination with antibiotics to control drug-resistant infections.

There is evidence that immunotherapy with mAbs synergistically improves outcomes in combination with antibiotics. In a recent study, Nielsen et al. developed a mAb against capsular carbohydrate on the bacterial surface and then assessed the efficacy of administering mAb treatment in combination with colistin; however, the C8 mAb, was able to detect only 60% of *A. baumannii* strains tested [11]. In this way, the mAb-based therapeutic approaches may be required to achieve sufficient strain coverage (~ 90% coverage) for empirical treatment against MDR, XDR, PDR models of *A. baumannii* infection. Antibodies directed against biomarkers may have the potentials to treat infectious diseases that threaten human health. Moreover, the application of antibodies does not affect the diversity of the host microbiota. In the future, therapeutic antibody preparation and injection into the live models may stimulate macrophages in conjunction with the serum complement system, leading to increased bacterial clearance and prevention of sepsis.

We developed a peptide-based mAb using a simple and reproducible method. The great advantage of this mAb, 3F10-C9, is its binding to OmpA and ability to identify *Acinetobacter*, as well as opsonization and cooperation in killing bacteria. As limitations of this study, we have not identified the detailed sequences of the OmpA in different pulsotypes of *A. baumannii* to confirm its conservation. Also, we have not evaluated the usefulness of such monoclonal antibody in an in-vivo model nor have we estimated the frequency of resistance to the mAb. Since the removal of OmpA and subsequent no expression of OmpA in the outer membrane has no effect on the survival of *A. baumannii* [51], specific antibodies against the OmpA cannot exert evolutionary pressure that accelerates resistance. Our 3F10-C9 mAb demonstrated the in-vitro practical applications including ELISA, Western blot, IFA, and opsonophagocytosis assays which may be appropriate to development of immunological tools required for *A. baumannii* research.

## Conclusion

Antibiotic-resistant *A. baumannii* has emerged as a major cause of healthcare-associated infections worldwide. As good companions of the antimicrobials or novel antibacterial compounds, validated antibodies could confer sufficient protection against *A. baumannii* infections and prevent the development of new drug resistance.

Our results highlighted the potentials of using OmpA as a conserved antigen among different *Acinetobacter* isolates. Future characterization of the conserved epitopes in order to further investigate the protective immunity against *A. baumannii* infection is highly desirable.

The produced 3F10-C9 mAb showed a good efficiency in detection, monitoring, and in the opsonization process which offers future applications and perspective on design and production of humanized antibodies for complementary protection. Further studies and more detailed evaluations are needed to perform more accurate and safer in-vivo experiments.

## Data Availability

All supporting data is available through the corresponding author and first author.

## Abbreviations

ELISA: Enzyme-linked immunosorbent assay
OmpA: Outer membrane protein A
IFA: Indirect immunofluorescence assay
KLH: Keyhole limpet hemocyanin
BSA: Bovine serum albumin
mAb: Monoclonal antibody
MDR: Multidrug-resistant
XDR: Extensively drug-resistant
PDR: Pandrug-resistant
KDa: Kilodalton, a unit of molecular mass equal to 1000 daltons
TMB: Tetra methyl benzidine
K_aff_: Affinity constant
PBS: Phosphate buffered saline solutions
HAT: Hypoxanthine/aminopterin/thymidine (HAT) medium
MBLs: Metallo-β-lactamases
OXA genes: A group of carbapenem-resistant OXA-type β-lactamases that have been identified in *A. baumannii*
PFGE: Pulsed-field gel electrophoresis
mM: Millimolar
RT: Room temperature
CFU: Colony-forming unit
DAB: 3,3′-Diaminobenzidine
